# Demonstration of physicochemical and functional similarity between Stimufend (pegfilgrastim-fpgk) and Neulasta (pegfilgrastim): A comparative analytical assessment

**DOI:** 10.1371/journal.pone.0309480

**Published:** 2024-10-24

**Authors:** Alison Sykes, Louise Ingram, Ulrich Kronthaler, Laurent Chevalet

**Affiliations:** Fresenius Kabi SwissBioSim GmbH, Eysins, Switzerland; University of Colorado Anschutz Medical Campus, UNITED STATES OF AMERICA

## Abstract

**Background:**

Pegfilgrastim is a long-acting recombinant human granulocyte colony-stimulating factor biologic that is indicated to reduce the incidence of infections, manifested by febrile neutropenia, in patients receiving myelosuppressive anti-cancer drugs and to increase survival in patients acutely exposed to myelosuppressive doses of radiation. Due to the high cost of biologic therapy and the scarcity of biosimilar alternatives, there is an unmet medical need for targeted biologics.

**Objective:**

This comparative analytical investigation aimed to confirm the similarity of biosimilar Stimufend^®^ (pegfilgrastim-fpgk) to reference product Neulasta^®^ (pegfilgrastim).

**Methods:**

The analysis was designed using state-of-the-art orthogonal techniques and side-by-side testing to compare the physicochemical and biological properties of these two products. The measured quality attributes included the primary structure and higher order structure of the molecule, purity/impurity profiles, product variants, process-related impurities, composition, content, and biological activity. The statistical analysis was based on risk ranking of the critical quality attributes (very low, low, moderate, high, very high), and scientific considerations in combination with the characteristics of the assay (sensitivity, selectivity, and variability). In addition, non-quantitative parameters were compared using a descriptive assessment of the product profile. Analytical similarity was concluded by quality attributes falling within the defined range of the originator product.

**Results:**

The results of this study confirm that Stimufend^®^ is biosimilar to Neulasta^®^ for all measured quality attributes. There are no clinically significant differences between Stimufend^®^ and Neulasta^®^, which was confirmed by the marketing approval for Stimufend^®^ by the Food and Drug Administration and the European Medicines Agency.

**Conclusion:**

The findings of this study provide robust evidence supporting the structural and functional biosimilarity between Stimufend^®^ and Neulasta^®^.

## Introduction

Biologics include a wide range of products, generated by using recombinant deoxyribonucleic acid (DNA) technology, which can provide targeted therapies for a variety of severe and life-threatening diseases that cannot be achieved using other approaches [1]. However, the high cost of treatment with biologics has resulted in an increasing challenge for health care systems worldwide [1].

A biosimilar is a biologic that meets extremely high standards for similarity to the originator biologic drug, also known as a reference product, and it is approved for use in the same indications. Biosimilars are developed to have the same quality, efficacy and safety as the reference product. They are intended to be less expensive alternatives to the originator products [2,3]. The globally accepted approach to determining biosimilarity to a reference product is through comparative analytical assessment [4–6].

The introduction of biosimilar products into the therapeutic landscape is made possible by, among other things, the implementation of comprehensive regulatory guidance. Europe (EU) has pioneered the regulation of biosimilar medicines by establishing a solid framework for their approval, shaping comparative analytical assessments globally. In 2006, the first biosimilar was approved by the European Medicines Agency (EMA) [2], with 86 more approvals since then [7]. The United States (US) followed suit in 2015 and has approved 33 biosimilars [8], 17 of which are cancer or cancer-related biosimilar products [9]. In the US, the Food and Drug Administration (FDA) created an abbreviated licensure pathway that enables biosimilar biological products to be licensed based on their similarity in structure and function to the reference product [10]. This provides an opportunity to bring more cost-effective agents to the market [11,12].

For obvious reasons, the biosimilar approval process is as rigorous as the process for any originator product. For a biosimilar to be approved by regulatory agencies, a developer must demonstrate highly similar quality, safety, efficacy, and potency to the reference product [1]. In addition, bioequivalence must meet certain criteria in terms of rate and extent of absorption, as outlined by the FDA guidance documents [13].

Pegfilgrastim is a long-acting recombinant human granulocyte colony-stimulating factor (G-CSF) biologic that is approved by the FDA [14] and the EMA [15]. It is indicated for subcutaneous use in patients with non-myeloid malignancies that are treated with cytotoxic myelosuppressive chemotherapy associated with a clinically significant incidence of febrile neutropenia, or in patients acutely exposed to myelosuppressive doses of radiation [15,16]. Pegfilgrastim stimulates the production of white blood cells, reducing the incidence and duration of neutropenia after chemotherapy [15]. This in turn decreases the incidence of infection, as manifested by febrile neutropenia, after receiving myelosuppressive anti-cancer drugs and increases survival in patients exposed to myelosuppressive doses of radiation [14].

The demand for pegfilgrastim biosimilar options is growing rapidly and has been facilitated by expedited approval processes. There are several approved pegfilgrastim biosimilars currently available on the market, including six approved or in development in the US [16] and a further 11 approved in countries outside of the US [17].

The clinical benefits of biosimilar innovation have been noted. Metanalysis data have indicated that G-CSF supportive care biosimilars show similar incidences of febrile neutropenia, time to absolute neutrophil count recovery, and duration of severe neutropenia compared to G-CSFs. This includes the incidence of bone pain, a common side effect of treatment with G-CSFs, as well as adverse drug events [18]. Others have noted that pegfilgrastim biosimilars have the potential to significantly reduce the cost of treatment for patients, payers, and the healthcare system [19].

The benefits of lower cost, fewer health care visits, and expanded treatment and administration options are becoming a reality [20]. Pegfilgrastim biosimilars are proving to be valuable in optimizing neutropenia management in cancer patients while improving health outcomes and tailoring cancer care [20].

This study is a comparative analytical investigation that compares the physicochemical properties, biological function, and potency of Stimufend^®^ (pegfilgrastim-fpgk, Fresenius Kabi Biosimilars GmbH) to Neulasta^®^ (pegfilgrastim, Amgen). Previous studies have been conducted on the safety and immunogenicity, and pharmacokinetics and pharmacodynamic equivalence, of Stimufend^®^ compared to Neulasta [21,22]. The objective of this comparative analysis was to assess the analytical similarity of Stimufend^®^ to Neulasta^®^.

## Materials and methods

**Fig 1:**
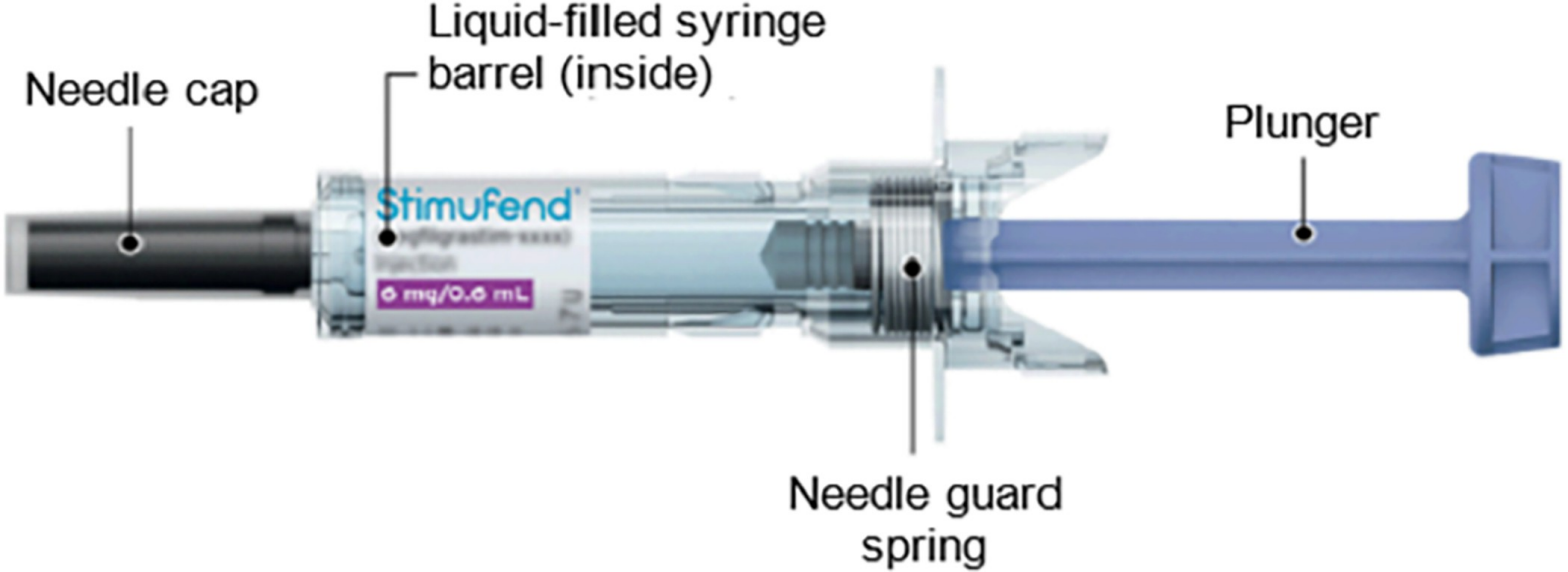
Illustration of Stimufend®. Stimufend® includes a needle cap, a liquid-filled syringe barrel inside the cartridge, a needle guard spring, and a plunger.

Stimufend^®^ is a sterile, preservative-free solution intended for subcutaneous injection. It is presented as a single-use, fixed-dose (6 mg pegfilgrastim-fpgk /0.6 mL solution), pre-filled syringe (Fig 1) [22].

Neulasta^**®**^ is a colorless solution for subcutaneous injection, or injection with an on-body device, presented in a pre-filled syringe containing 6 mg of pegfilgrastim in 0.6 mL solution [15].

The batch formulation of Stimufend^®^ was based on the Neulasta^®^ formulation both at pH4.0 with the composition, listed in Table 1.

**Table 1:** Neulasta^®^ and Stimufend^®^ formulations.

	Neulasta^®^ 6 mg solution for injection	Stimufend^®^ 6 mg solution for injection
Component	Quantity per 0.6 mL	Quantity per 0.6 mL
Pegfilgrastim	6.0 mg	6.0 mg
Sorbitol	30.0 mg	30.0 mg
Sodium	0.02 mg	-
Acetate	0.35 mg	-
Sodium Acetate Trihydrate	-	0.12 mg
Glacial Acetic Acid	-	0.30 mg
Polysorbate 20	0.02 mg	0.02 mg

To ensure that Stimufend^®^ met high-quality standards, its manufacturing process was developed with defined manufacturing procedures, critical process parameters, in-process controls, and release specifications. A quality profile that matched the quality target product profile (QTPP) was developed during the design of the manufacturing process resulting in the identical pharmaceutical form, concentration, composition, and route of administration for Stimufend^®^ when compared to Neulasta^®^.

The developed process matched or exceeded the removal of process-related impurities such as host cell DNA, host cell proteins and sodium cyanoborohydride compared to the originator and complied with the latest regulatory recommendations. Solution composition was assessed to determine the polysorbate (PS) 20 and sorbitol content. Composition attributes were considered low (PS 20) to very low (sorbitol) criticality. The formulation was created to replicate the original product and was verified to align with the product’s shelf life and degradation profile. This was part of the comprehensive overall evaluation of composition and similarity and was controlled during the manufacturing process.

To assess the similarity of the product quality, comparative analytical assessments of Neulasta^®^ and Stimufend^®^ were conducted. Samples were tested using methods either validated following the International Council for Harmonization guidelines for method validation (ICH Q2) or qualified as fit-for-purpose.

In total, 28 US-licensed and 24 EU-approved Neulasta^®^ lots were used for the comparative analytical assessment of similarity with Stimufend^®^ (16 lots). State-of-the-art orthogonal techniques, with side-by-side testing as appropriate, were used to provide independent confirmation, measure similar attributes, and compare physicochemical properties. Orthogonal techniques refer to a set of methods that are independent of each other and characterize quality attributes via an alternative methodology to provide complementary information to broaden the depth of understanding of the product. These techniques operate on different aspects of quality attributes and are not dependent on the results obtained from other techniques. Using a range of orthogonal techniques facilitated the investigation of the protein from different perspectives and ensured the sensitivity of the similarity assessment in detecting minor potential differences.

The quality attributes included the primary structure and higher order (secondary and tertiary) structure of the molecule, purity/impurity profiles, product variants, process-related impurities, composition, content, and biological activity. These quality attributes were determined by a quality-by-design approach, derived from the identification of the product’s critical quality attributes, and based on the risk of the potential impact on activity, pharmacokinetic (PK), pharmacodynamics (PD), safety, efficacy, and immunogenicity (Table 2). The extinction coefficient of 0.86 (mg/mL)^-1^cm^-1^ was used to characterize the composition of the molecule. The functional properties were evaluated by relative potency (proliferation activity), absolute binding kinetic, and affinity to granulocyte colony-stimulating factor receptor (G-CSF-R). Internal cross-quantified reference standards were used to calculate drug potency and used in every analytical session for all methods to allow a bridge for analytical assessment.

**Table 2:** Physicochemical and functional quality attributes and methods used in the assessment.

**Method**	**Purpose**
**Primary Structure**	
Peptide Mapping by LC-MS/MS (R)	Sequence confirmation, PTMs and mPEG attachment site
Intact Mass by MALDI-TOF	Determination of MW of Pegfilgrastim & Dimer
Polydispersity LC-MS	Determination of MW and PDI
**Higher Order Structure**	
Peptide mapping by LC-MS/MS (NR)	Identification of disulfide bridges and free cysteine
Ellman’s assay	Determination of levels of free thiols
Near & Far-UV CD	Secondary and tertiary structure by near and far UV CD
Fluorescence spectroscopy	Emission spectra at 257, 274 and 295 nm
Thermal analysis by nanoDSC	Determination of thermal transition temperatures
NMR	Comparison of primary, secondary and tertiary structure
**Purity & Impurities**	
SE-HPLC	Determination of levels monomer, aggregates, HMW/Di-pegylated and free G-CSF
AUC	Determination of levels of Monomer and dimer, oligomers and sedimentation coefficients
SEC-MALS	Determination of MW of conjugate, protein, mPEG and Hydrodynamic radius
SVP by Light Obscuration	Determination of particles in size ranges from 2μm to > 25 um
CE-SDS (reducing)	Determination of % purity and % LMW
Free G-CSF by RP-HPLC	Determination of % free G-CSF
Free mPEG RP-HPLC ELSD	Determination of levels of free mPEG
**Product Variants**	
icIEF	Analysis of charge variant distribution, acidic and basic clusters
SCX-HPLC	Analysis of charge variant profile, pre-/post main peak clusters
RP-HPLC	Determination of levels of hydrophobicity variants
**Content**	
UV absorbance at 280 nm based on protein content obtained by AAA	Establishment of extinction coefficient
Protein Concentration by UV280	Determination of protein concentration
Extractable Volume	Determination of extractable volume
**Biological Activity**	
G-CSF-R Binding Affinity by SPR	Measurement of binding affinity to G-CSF-R, ka, kd and KD
Potency by M-NFS-60 Proliferation	Measurement of relative potency and calculation of specific activity

AAA, amino acid analysis; AUC, analytical ultracentrifugation; CD, circular dichroism; CE-SDS, capillary gel electrophoresis-sodium dodecyl sulfate; DSC, differential scanning calorimetry; ELSD, evaporative light scattering detection; ET, equivalence test; G-CSF, granulocyte colony-stimulating factor; G-CSF-R, granulocyte colony-stimulating factor receptor; HMW, high molecular weight; HPLC, high-performance liquid chromatography; icIEF, imaged capillary iso electric focusing; KD, equilibrium dissociation constant or affinity constant; LC-MS/MS, liquid chromatography mass spectrometry/molecular weight; LMW, low molecular weight; MALDI-TOF, matrix assisted laser desorption ionization time-of-flight; mPEG, monomethoxy poly (ethylene glycol); MW, molecular weight; NMR, nuclear magnetic resonance; NR, non-reduced; PDI, polydispersity index; QR, quality range; RD, visual comparison of raw data; RP-HPLC, reverse phase high-performance liquid chromatography; SCX, strong cation exchange; SDS, sodium dodecyl sulfate; SDS-PAGE, sodium dodecyl sulfate polyacrylamide gel electrophoresis; SE-HPLC, size-exclusion high performance liquid chromatography; SEC MALS, size-exclusion chromatography multi-angle laser light scattering; SPR, surface plasmon resonance; SVP, sub-visible particles; UV, ultraviolet.

Evaluation of the comparative analytical data was performed taking into consideration the criticality risk ranking (very low, low, moderate, high, very high) together with the nature, distribution, abundance, and sensitivity of the assay for the attribute and the quantitative or qualitative nature of the measurement with reference to the FDA and EMA guidance for industry [2,14,23,24]. For attributes of “very high” criticality, i.e., the highest degree of importance, equivalence testing (ET) was conducted.

Where data were amenable, they were subjected to statistical analysis. The QR limits were set based on the range of the values acquired for reference product variation, defined as X times Standard Deviation (SD). If 90% of the data points were within the QR, high similarity was said to have been proven. For high criticality attributes a quality range was set using a multiplier x = 2 to ensure a robust assessment of similarity using a different statistical approach to the ET. In the evaluation of quality attributes with a “high” to “moderate” criticality score, a QR approach with a multiplier x = 3 was used. These analysis multipliers are factors to adjust the control limits based on the size of the data and level of confidence, thereby ensuring that the control limits are appropriately rigorous and conservative.

### Primary and higher order structure

The primary structure was assessed using peptide mapping by liquid chromatography mass spectrometry in reduced condition (LC-MS [R]), amino acid analysis, intact mass by matrix-assisted laser desorption ionization time-of-flight (MALDI-TOF), and polydispersity by LC-MS. The higher order structure, including secondary and tertiary structure, was assessed using peptide mapping by LC-MS/MS (non-reduced [NR] for detecting expected two disulphides), Ellman’s assay (for detecting free cysteines including Cys18), near and far-ultraviolet (UV) circular dichroism (CD), fluorescence spectroscopy, thermal analysis by nanoDSC, and nuclear magnetic resonance (NMR). NMR spectroscopy was employed to confirm the structure and type of polyethylene glycol (PEG) linkages as well as to verify the sites of PEGylation. The assessment of the primary structure included the measurement of the sequence confirmation, the post-translational modifications (PTMs) levels, the monomethoxy poly (ethylene glycol) (mPEG) attachment site, molar absorptivity, and the molecular weight (MW) of pegfilgrastim, dimer, mPEG and polydispersity.

### Purities and impurities

The degree of purity and impurities were evaluated with methods including size-exclusion high-performance liquid chromatography (SE-HPLC), analytical ultracentrifugation (AUC), size-exclusion chromatography-multi-angle laser light scattering (SEC-MALS). To detect low MW impurities, capillary gel electrophoresis-sodium dodecyl sulfate (CE-SDS) was used, while sub-visible particles were determined by light obscuration.

Furthermore, to determine levels of free mPEG and G-CSF, as a result of process residuals or degradation of the pegylation cross-linkage, reverse phase HPLC (RP-HPLC) with evaporative light scattering detection (ELSD) and RP-HPLC with UV detection were used respectively.

### Product variants

Product variants were determined using imaged capillary isoelectric focusing (icIEF) and strong cation exchange-HPLC (SCX-HPLC) for charged variants and RP-HPLC assay for oxidized and other product variants.

### Composition and content

Composition analysis of PS20 used the ELSD-HPLC assay and the refractive index HPLC (RI-HPLC) assay was used to determine sorbitol content. The content analysis included amino acid analysis (AAA), UV molar absorptivity for absorbance at 280 nm based on protein content obtained by AAA, protein concentration by absorbance at 280nm, and extractable volume.

### Biological activity

Biological activity was measured using G-CSF-R binding affinity by surface plasma resonance (SPR), and potency by M-NFS-60 cell proliferation assays.

### Specifications

The methods used and analysis approach followed the ICH Q6B specifications: Test Procedures and Acceptance Criteria for Biotechnological/ Biological Products and FDA Guidance for Industry on Analytical Procedures and Methods Validation for Drugs and Biologics (July 2015). The test items included in the specification were selected using the European Pharmacopoeia (Ph. Eur.) and United States Pharmacopoeia (USP) guidelines.

## Results

The results of the comparative analytical assessment are summarized in Table 3.

**Table 3:** Comparative analytical results.

Clinical relevance	Level of criticality	Similarity analysis	Assay	Measurement				Stimufend^®^ vs Neulasta^®^
								(Equivalence^a^ /% within QR^b^/ RD)
**Primary structure**								
Efficacy, Safety & Immunogenicity	High	RD	LC-MS/MS (R)	Sequence confirmation				Identical
	Moderate			Comparison of levels of PTM				Similar
	Low			mPEG attachment site				Similar
	High	RD	Amino Acid Analysis	Determination of protein concentration				Similar
				Molar absorptivity				Similar
	Moderate	RD	MALDI-TOF	Pegfilgrastim MW				Minor differences. Not clinically meaningful
				Dimer MW				Minor differences. Not clinically meaningful
	Very low	RD	LC-MS	Polydispersity				Similar
**Higher order structure**								
Efficacy, Safety & Immunogenicity	High	RD	LC-MS/MS (NR)	Disulfide Bonds Free Cys18 Cys37-Cys43 Cys65-Cys75				Similar Similar Similar
		QR	Ellman’s	Comparison of the amount of free sulfhydryl groups (mol/mol)				100%
		QR	Nano DSC	Thermal Transition Temperatures °C			Tm 1:	100%
							Tm 2:	100%
		QR	Fluorescence Spectroscopy	Comparison of Secondary structure			257 nm	100%
							274 nm	100%
							295 nm	100%
		RD	Circular Dichroism	Near UV				Similar
				Far UV				Similar
		RD	NMR	1D ^1^H spectra				Similar
		RD	NMR	2D ^1^H-^15^N SO-FAST HMQC spectra				Similar
	Moderate	QR	SE-HPLC	Determination of aggregate content and monomeric purity	%Monomer			36% Stimufend^®^ higher
					%Aggregates			91%
					%Di-Peg/HMW			45% Stimufend^®^ lower
					%Free G-CSF			100%
	Moderate	RD	AUC	Determination of aggregate/mono -meric content	%Monomer			Similar
					%Dimer			Similar
					Monomer S			Similar
					Dimer S			Similar
	Moderate	RD	SEC-MALS	Size heterogeneity	MW Conjugate, kDa			Similar
					MW protein, kDa			Similar
					MW PEG, kDa			Similar
					Hydrodynamic radius nm			Similar
	Moderate	RD	PAMAS (Low volume Light Obscuration)	Comparison of the numbers of sub-visible particles/mL	No. of Particles >25 μm/ml			Similar
					No. of Particles >10 μm/ml			Similar or Stimufend^®^ lower
					No. of Particles >5 μm/ml			Similar or Stimufend^®^ lower
					No. of Particles >2 μm/ml			Similar or Stimufend^®^ lower
Efficacy	Moderate	RD	Reduced CE-SDS	Determination of electrophoretic mobility and purity	%Purity			Similar or Stimufend^®^ higher
					%LMW			Similar or Stimufend^®^ lower
Efficacy	Moderate	RD	Free G-CSF by RP-HPLC	Determination of free G-CSF content	%Free G-CSF			Similar all values <LOQ
None	Very Low	RD	RP-HPLC with ELSD detection	Determination of Free mPEG content	Free mPEG (μg/mL)			Similar or Stimufend^®^ lower
**Product Variants**								
Efficacy	Moderate	RD	icIEF	Comparison of isoelectric point(s)	pI Cluster 1			Similar
					pI Cluster 2			Similar
					pI Cluster 3			Similar
					pI Cluster 4			Similar
					pI Cluster 5			Similar
				Comparison of % of each cluster	% Cluster 1			Similar
					% Cluster 2			Similar or Stimufend^®^ lower
					% Cluster 3			Similar
					% Cluster 4			Similar or Stimufend^®^ higher
					% Cluster 5			Similar
					% Total cluster 4+5			Similar or Stimufend^®^ higher
					Cpl% of cluster 4			Similar
	Moderate	RD	SCX-HPLC	Comparison of charge variant distribution	%Total pre-peaks			Similar or Stimufend^®^ lower
					%Main peak			Similar or Stimufend^®^ higher
					%Total post-peaks			Similar Stimufend^®^ higher but <1%
					%Total impurities			Similar or Stimufend^®^ lower
Efficacy & PK	Moderate	QR	RP-HPLC	Comparison of hydrophobicity variants	Main peak			77% Stimufend^®^ higher
					%Total Ox.			77% Stimufend^®^ higher but Levels of Total Ox <2%
					%Total Red./Deamidated			62% Stimufend^®^ lower
**Content**								
Pharmacokinetics & Efficacy	Very high	QR ET	Protein concentration (UV280)	Protein concentration (UV280)		Protein concentration (mg/mL)		92%^c^ Passed Equivalence Test
		QR	Extractable volume (Ph. Eur. 2.9.17, USP<1>)	Extractable volume (mL)				100%
		QR	Calculated	Total extractable protein content (mg)				100%
**Biological Activities**								
Mechanism of Action & Efficacy	Very high	RD	G-CSF-R Binding SPR	ka (x10^4^ 1/Ms)				Similar
		RD		kd (x10^-5^ 1/s)				Similar
		QR		KD (pM)				100%
		QR ET	Pegfilgrastim induced MNFS- 60 cell proliferation	Relative potency (%EC50)				100% Passed Equivalence Test
		QR ET		Specific activity (x10^6^ IU/mg)				100% Passed Equivalence Test

AUC, analytical ultracentrifugation; CE-SDS, capillary gel electrophoresis-sodium dodecyl sulfate; DNA, deoxyribonucleic acid; DSC, differential scanning calorimetry; ELSD, evaporative light scattering detection; ET, equivalence test; G-CSF, granulocyte colony-stimulating factor; G-CSF-R, granulocyte colony-stimulating factor receptor; HPLC, high-performance liquid chromatography; icIEF, imaged capillary iso electric focusing; ka, association constant; kd, disassociation constant; KD, equilibrium dissociation constant or affinity constant; LC-MS/MS, liquid chromatography mass spectrometry/molecular weight; LMW, low molecular weight; LOQ, limit of quantitation; MALDI-TOF, matrix assisted laser desorption ionization time-of-flight; mPEG, monomethoxy poly (ethylene glycol); MW, molecular weight; NMR, nuclear magnetic resonance; NR, non-reduced; QR, quality range; RD, visual comparison of raw data equivalent to descriptive assessment; RP-HPLC, reverse phase high-performance liquid chromatography; SCX, strong cation exchange; SDS, sodium dodecyl sulfate; SE-HPLC, size-exclusion high performance liquid chromatography; SEC MALS, size-exclusion chromatography multi-angle laser light scattering; SPR, surface plasmon resonance; UV, ultraviolet.

^*a*^*ET was determined as 1*.*5σR of Neulasta data and results were determined as 90% CI of mean difference between two products*.

^*b*^*The QR limits were set based on the range of the values obtained for US reference product variation*, *expressed as Mean +/-Standard Deviation (SD)*

X times SD. High similarity was considered to have been demonstrated if 90% of data points were within the QR. X = 3 unless otherwise indicated.

### Primary structure

**Fig 2:**
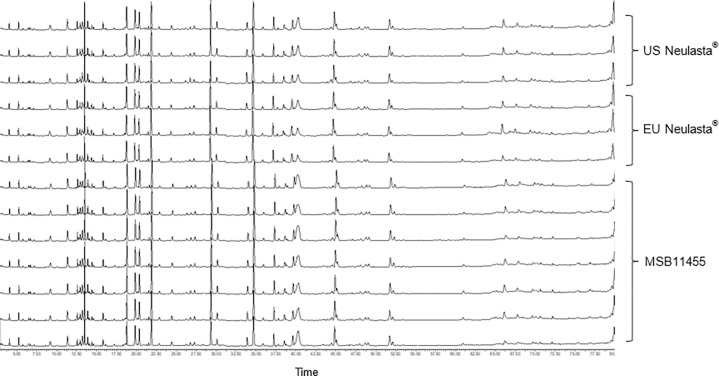
Peptide mapping of US and EU Neulasta^®^ and MSB11455 (Stimufend^®^) batches. Combined with LC-MS/MS data (not shown) the peptide mapping profiles support the conclusion that Stimufend^®^ has an identical amino acid sequence to the US and EU reference drugs.

The results indicated that Stimufend^®^ and Neulasta^®^ were identical in primary sequence with similar PTM levels, mPEG attachment site, molar absorptivity, and polydispersity. Minor, clinically non-significant, differences were noted in pegfilgrastim MW and dimer MW by MALDI-TOF analysis (Fig 2).

### Higher order structure

The purpose of assessing the higher order structure was to compare the secondary and tertiary structure and confirm the similarity of protein conformation by identifying disulfide bridges and free cysteine, levels of free thiols, tertiary structure, fluorescence emission spectra at 257, 274 and 295 nm, near and far UV CD spectrums and thermal transition temperatures.

The similarity analysis indicated that disulfide bonds Cys37-Cys43, Cys65-Cys75, and free Cys18, were similar between Stimufend^®^ and Neulasta^®^, as were the free sulfhydryl groups. Additionally, thermal transition temperatures using nano differential scanning calorimetry and the comparison of maximum emission wavelengths using fluorescence spectroscopy were 100% within the QR demonstrating similar tertiary structures.

**Fig 3:**
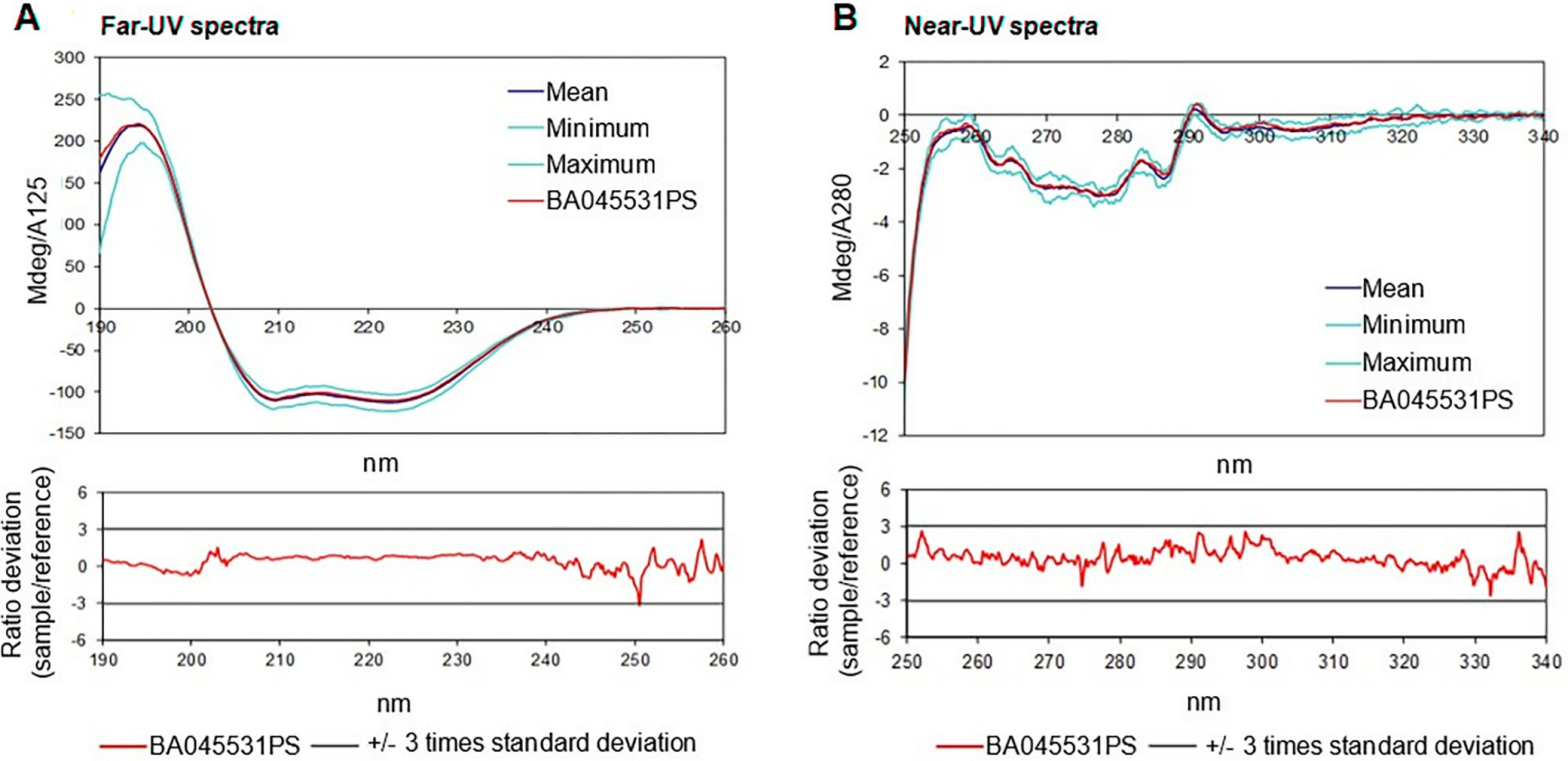
High order structure analysis by circular dichroism. Representative far-UV and near-UV CD spectra of MSB11455 and reference pegfilgrastim suggest similar secondary and tertiary structures. (A) Secondary structure measured by far-ultraviolet circular dichroism spectra. (B) Tertiary structure measured by near-ultraviolet circular dichroism spectra.

Comparison of far-UV CD spectra show comparable signals in the 190-230nm region demonstrating similar secondary structures while near-UV CD spectra demonstrate the same minima and maxima typical for aromatic amino acids indicating the same microenvironment and hence comparable tertiary structures. The visual comparability was further confirmed by determining a weighted spectral difference plot showing that Stimufend^®^ is within a mean range determined for Neulasta^®^. The near-UV and far-UV spectra are provided in the accompanying graphic (Fig 3) with their associated ‘mean spectrum’ plot below.

### Purity and impurities

The purpose of the purity and impurity tests was to determine the nature and amount of high MW species relative to the major monomeric form, levels of free G-CSF, protein and mPEG hydrodynamic radius to compare the size of the HMW impurities, particle size ranges from 2μm to > 25 μm, % purity and % low MW, and levels of free mPEG.

**Fig 4:**
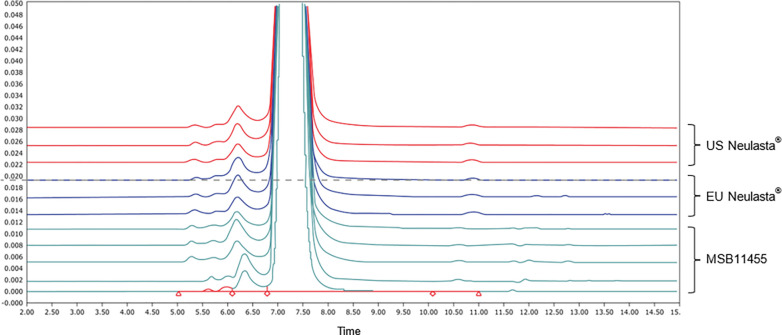
Purity profile for Stimufend^®^ and Neulasta^®^. The plot shows overlaid size exclusion–HPLC profiles of US-licensed Neulasta^®^, EU-approved Neulasta^®^, and MSB11455 batches, and a similar profile for Stimufend^®^ compared to Neulasta^®^.

The results showed that Stimufend^®^ was similar in aggregate/monomeric content, size heterogeneity, number of sub-visible particles per mL, free G-CSF and mPEG content, and electrophoretic mobility and purity when compared to Neulasta^®^. Chromatograms, as illustrated in Fig 4, provide a visual representation of the elution pattern and molecular size distribution of sample components, essential for assessing purity profiles for monomeric and HMW species.

**Fig 5:**
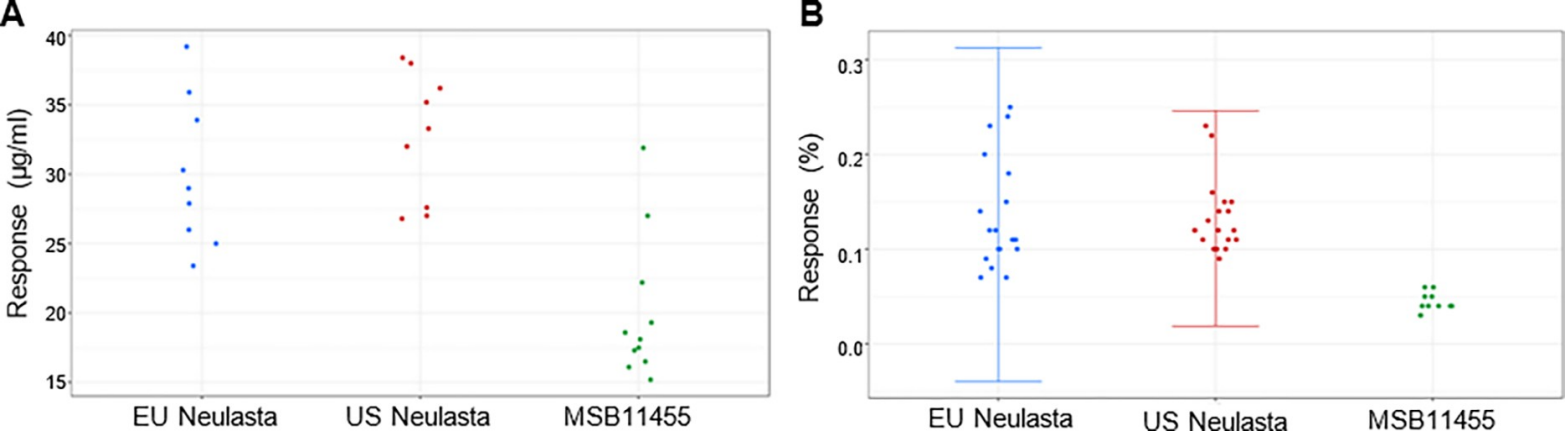
Purity levels for Stimufend^®^ and Neulasta^®^. Higher purity levels were noted for Stimufend^®^ compared to Neulasta^®^ using reversed phase high-performance liquid chromatography and size exclusion chromatography high-performance liquid chromatography. (A) Reversed phase high-performance liquid chromatography of free monomethoxypolyethylene glycol with evaporative light scattering detection (μg/ml). (B) Size exclusion high-performance liquid chromatography of free granulocyte colony-stimulating factor (%). Quality range error bars of mean ±3 standard deviations.

Stimufend^®^ showed a higher percentage of monomer (and therefore fewer impurities) with lower Di-Peg/HMW compared to Neulasta^®^, with 36% and 45% falling within the QR respectively. Similar levels of the remaining impurities by SE HPLC were determined with 91% of Stimufend^®^ aggregates and 100% of free G-CSF within the QR (Table 3 and Fig 5B for residual GCSF). In addition, lower levels of residual mPEG were detected in Stimufend^®^ which together with the previous data suggests an overall lower impurity profile (Fig 5A).

### Product variants

The variant analysis plays a crucial role in ensuring product quality, safety, and consistency. The product variant analysis here used icIEF, SCX-HPLC, and RP-HPLC assays to compare isoelectric points, charge variant distribution, acidic and basic clusters, oxidized variant profile, pre-/post main peak clusters and total impurities and levels of hydrophobic variants.

The isoelectric points, percentage of each cluster, charge variant distribution, and hydrophobicity variants were similar between Stimufend^®^ when compared to Neulasta^®^. In measurements where Stimufend^®^ showed higher levels (charge variant distribution and the comparison of hydrophobicity variants), it was no more than 2% which was shown to have no impact on activity at these low levels.

### Content

The goal of the content assessment was to determine protein content and concentration and establish the extinction coefficient and the extractable volume to ensure an equivalent dose would be delivered. The comparison analysis indicated that 100% of the extractable volume and total extractable protein content fell within the QR when comparing Stimufend^®^ to Neulasta^®^. For highly critical attributes it was also recommended by the FDA to perform equivalence testing. Hence protein concentration was further evaluated, and statistical equivalence of the means was confirmed.

### Biological activity

**Fig 6:**
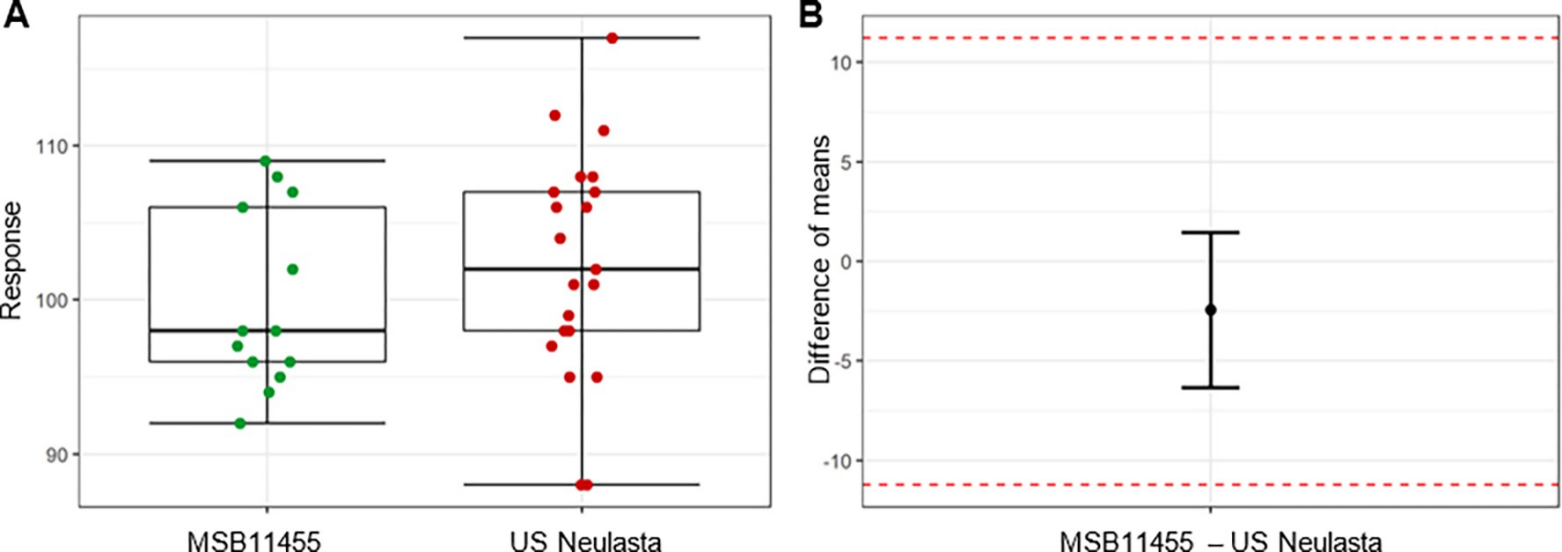
Pegfilgrastim-induced M-NFS-60 cell proliferation by fluorescence, relative potency (%) for Stimufend^®^ and Neulasta^®^. (A) Data distribution. (B) Equivalence test.

The biological activity assessment aimed to determine binding affinity by SPR to G-CSF-R (association and dissociation constants [ka and kd] and equilibrium dissociation constant [KD]) as well as relative potency and specific activity. Analysis was performed on raw data using a QR approach and as described for content, relative potency and specific activity being highly critical attributes were also evaluated for equivalence. Stimufend^®^ relative potency and specific activity were highly similar to Neulasta^®^ and all lots were within the QR and were shown to have equivalent means. Fig 6 provides the equivalence test results for the relative potency clearly showing the close agreement between the distributions and therefore similarity in potency.

**Fig 7:**
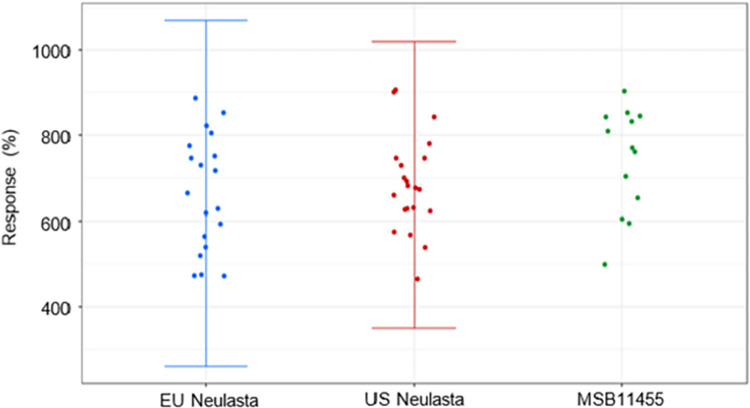
Binding affinity of Stimufend^®^ and Neulasta^®^. Binding to granulocyte colony-stimulating factor receptor by surface plasmon resonance. Similar granulocyte colony-stimulating factor receptor binding was found between Stimufend^®^ and Neulasta^®^. Quality range error bars of mean ±3 standard deviations.

The examination revealed that the interaction of G-CSF-R with SPR showed a comparable pattern for Stimufend® and Neulasta®, with all KD values falling within the QR range (Fig 7).

## Discussion

The development of biosimilars depends on thorough structural and functional characterization [5]. The specifications for the level of comparability between biosimilar therapeutics and their reference products are stringent, and the final product must be shown to be highly analytically similar based on a set of quality attributes [2,25]. This is true for all biosimilars; here we present pegfilgrastim.

The results of this comparative analysis successfully demonstrated that Stimufend^®^ was highly similar to Neulasta^®^. Any differences in quality attributes were justified and no clinically meaningful differences between Stimufend^®^ and Neulasta^®^ were identified.

Of importance in this analysis, compliance with both FDA and EMA regulations was necessary with the utilization of both US-licensed (reference product [RP]) and EU-approved (Reference Medicinal Product [RMP]) comparators. Critically, the FDA and EMA (hereafter referred to as agencies) have their specific requirements and guidelines for assessing statistical comparability in the CMC analysis of pharmaceutical products, including biologics and biosimilars [26]. While there are common principles, differences and nuances exist between the two agencies. For example, the EMA mandates that biosimilars must demonstrate similarity in terms of ’quality, efficacy, and safety,’ while the FDA emphasizes ’safety, purity, and potency’ as their criteria [26].

The agencies share a focus on rigorous analytical and statistical comparability assessments to demonstrate biosimilarity, but the specific methods and statistical approaches used may vary. For the FDA, the emphasis is on the concept of "totality of the evidence" and requires the reference product to be a licensed U.S. product [26], for the EMA there may be more flexibility in selecting the reference product if appropriately justified.

The major complexities that were overcome in this analysis were the selection of reference products that satisfied both agencies’ criteria and the adaptation of analytical strategies to meet the distinct expectations of each agency.

Harmonizing these requirements and expectations of the FDA and EMA for biosimilar development necessitated careful planning, resource allocation, and a deep understanding of each agency’s unique regulatory landscape. Successful alignment with both agencies ultimately led to broader market access, however, it required a strategic and well-coordinated approach.

Within the realm of biosimilar development, another critical facet is the rigorous examination of statistical comparability criteria and quality attributes. This involves scrutinizing how these criteria are perceived and adopted by various agencies worldwide [27,28]. The approach to these criteria can exhibit dynamic evolution across different regions and differences in regulatory standards and guidelines. Region-specific requirements and updates in regulatory guidelines can result in variations in how the RP and RMP are assessed [29]. Additionally, variability can arise from differences in the sources of the RP and RMP, distinct analytical methods employed by the agencies, and variations in data collection, analysis, and interpretation methodologies [28]. Despite this, our comprehensive analysis unequivocally demonstrated a remarkable similarity to the RMP and RP, reflecting a concerted effort to align with global regulatory standards, thereby reinforcing the biosimilar’s reliability and therapeutic equivalence.

Analytical techniques tailored for pegylated proteins played a pivotal role in the success of this analysis [28]. These techniques were specifically designed to decipher the unique structural complexities of pegylated proteins, presenting distinctive challenges in their characterization [30]. In this intricate landscape, our scientific approach leveraged cutting-edge methodologies, such as advanced mass spectrometry, circular dichroism spectroscopy, and specialized NMR techniques. These analytical advancements empowered us to unravel the structural intricacies of pegylated proteins, ensuring their biosimilarity was established with precision. Many of these techniques have been used successfully in similar studies [5,6,31].

Analyzing pegylated proteins in the context of biosimilar development posed unique challenges. The covalent attachment of PEG can introduce heterogeneity, making it difficult to determine the specific sites and lengths of PEG chains [32]. This heterogeneity can induce structural changes and alter the protein’s size and charge, complicating its characterization [33]. Accurate quantification of PEGylation and the ratio of pegylated to non-pegylated protein was therefore critically important and included in our analysis.

Furthermore, assessing the impact of PEGylation on the protein’s biological activity and pharmacokinetics required specialized bioassays [32]. Overcoming these complexities necessitated the use of advanced analytical techniques, as previously mentioned, to gain a comprehensive understanding of the structural and functional characteristics of pegylated proteins. Comprehensive quality control processes were paramount in addressing these complexities and ensured the effective comparison of products.

Our biosimilarity pursuit is characterized by a thorough exploration of statistical comparability criteria, meticulous management of variability, and the skillful application of specialized analytical techniques designed for pegylated proteins. These considerations collectively underscore our unwavering commitment to delivering biosimilars of the highest quality and efficacy.

The findings of this study contribute to the current global landscape of biosimilar research and provide robust evidence supporting the structural and functional biosimilarity between Stimufend^®^ and Neulasta^®^.

## Conclusion

This comparative analytical investigation compared the analytical similarity of Stimufend^®^ to Neulasta^®^. The results indicate a high degree of similarity. There were no clinically significant differences noted between Stimufend^®^ and Neulasta^®^ across various critical attributes, and any changes in quality characteristics could be explained and justified, supporting the biosimilarity of Stimufend^®^ to the reference product, Neulasta^®^. These findings are crucial for demonstrating the safety and efficacy of Stimufend^®^ as a biosimilar alternative.
